# Regulating glycolysis and heat shock proteins in Gannan yaks (*Bos* *grunniens*) in response to hypoxia of the Qinghai–Tibet Plateau

**DOI:** 10.5194/aab-64-345-2021

**Published:** 2021-08-19

**Authors:** Yuliang Wen, Jiqing Wang, Xiu Liu, Shaobin Li, Jiang Hu, Yuzhu Luo

**Affiliations:** Faculty of Animal Science and Technology, Gansu Agricultural University, Gansu Key Laboratory of Herbivorous Animal Biotechnology, Lanzhou 730070, China

## Abstract

Glycolysis and heat shock proteins (HSPs) play an important role in hypoxia-intolerant species during hypoxia conditions. This study was conducted to evaluate the differences of glycolysis and heat shock proteins (HSPs) in Gannan yaks (*Bos grunniens*), with the main goal of understanding how the response to hypoxia changes with altitude. Here, the genes and enzymes of glycolysis and HSPs were detected in heart, liver, lung, kidney, and longissimus dorsi from Gannan yaks at different altitude (2500 and 3500 m) using qPCR, western blot, and enzyme kits. The results showed that the expression of *HIF1A* and *PDK4* was increased with altitude (P<0.01) in above tissues. Significantly increased lactate dehydrogenase (LDH), adenosine triphosphate (ATP), and nicotinamide adenine dinucleotide (NADH) levels and the ratio of NADH/NAD${}^{+}$ were also observed in heart, lung, and longissimus dorsi tissues (P<0.05), as well as a decreased citric acid (CA) level (P<0.05). Furthermore, we observed significant global increases in the protein and mRNA expression levels of both the ATP-independent HSP27 and the ATP-dependent HSP60 during hypoxic conditions (P<0.01). These findings revealed that hypoxia-reprogrammed glucose metabolism promotes energy supply via up-regulated glycolysis and weakness of the tricarboxylic acid (TCA) cycle. HSPs were activated and the prioritization of cytoprotective protein chaperone functions over energy conservation in yak under hypoxic conditions. These results are useful to better understand the unique adaptability of yak, allowing them to survive in hypoxia conditions.

## 1 Background

The Qinghai–Tibet Plateau is located in the interior of Asia, with an average elevation above 4000 m, making it the highest plateau in the world. Previous studies have found that oxygen content at an altitude of 4000 m is only 60 % of that at sea level (Beall, 2007). Reduced oxygen partial pressure can lead to insufficient oxygen supplies in bodily tissues, affecting the normal physiological functions of animals (Stuart et al., 2019).

The yak (*Bos grunniens*) is a symbolic animal in Tibet and has already evolved systemic and cellular adaptations to high-altitude environments (between 2000 and 5000 m) on the Qinghai–Tibetan Plateau (Qiu et al., 2012; Mcclelland and Scott, 2019; Ding et al., 2020). Gannan yak is a local yak breed that can adapt to the plateau low-oxygen environment formed after long-term natural and artificial selection. It is mainly distributed in Hezuo City and Maqu County (Gansu, China). In China, the yak population is estimated to exceed 14 million animals, serving as a source of basic resources (such as meat, milk, dung for fuel, and hides for tents) for Tibetans and other nomadic pastoralists who inhabit hypoxia environments (Wiener et al., 2010).

Under a hypoxic environment, cellular aerobic respiration can become severely compromised, impeding adenosine triphosphate (ATP) production and disrupting the cellular energy balance (Hochachka, 1986). Simultaneously, hypoxia can contribute to a loss of protein integrity and structural damage due to disruptions in the redox balance, especially among mitochondrial proteins (Kaufman et al., 2017). To avoid this outcome, glucose metabolism and unfolded-protein response (UPR) were changed to maintain energy supply and proteostasis under hypoxic conditions (Pilkis and Granner, 1992; Hetz and Papa, 2018). The UPR consists of two primary components: the ubiquitin–proteasome system and molecular chaperones, also known as heat-shock protein (HSPs). HSPs can be classified according to their functions, as holdases, foldases, or disaggregases, and are typically classified according to their molecular weights, such as HSP-27, HSP-40, HSP-60, HSP-70, and HSP-90 (Díaz et al., 2015).

Currently, few studies have examined the HSP response to hypoxia in yak, which represents a significant gap in our knowledge. Understanding the roles played by glycolysis and HSPs during energy metabolism and the cytoprotective adaptations to hypoxia in yak is important. We explored changes in the mRNA expression levels of hypoxia-inducible factor 1α (*HIF1A*) and pyruvate dehydrogenase kinase 4 (*PDK4*), the gene and protein expression levels of HSP27 and HSP60, and measured the contents of lactate dehydrogenase (LDH), ATP, nicotinamide adenine dinucleotide (NADH/NAD${}^{+}$), and citric acid (CA) in heart, lung, and longissimus dorsi tissues obtained from both 2500 and 3500 m Gannan yaks. The detection of significant altitude-related changes would help to understand how the response to hypoxia changes with altitude and could provide important information regarding ways to prevent hypoxia-related sickness both in animals and in people.

**Table 1:** Primers used in the present study.

Gene	GenBank ID	Primer sequence (5${}^{\prime }\to$3${}^{\prime }$)	Product size (bp)	$T_{m}$ / ${}^{\circ }C$	Application
*HIF1A*	XM_005890693	F: TCAAGCAGTAGGAATTGGAACA R: GTGATGTGGTAGTTGCACGA	62	59	qPCR
*PDK4*	XM_005888179.2	F: GAGCATTTCTCGCGCTACAG R: TGCGTTTTCTGAACCGAAGTC	72	60	
*HSP27*	NM_001025569.1	F: TGGAGATCACTGGCAAGCAC R: ATTTGCGAGTGAAGCAACGG	72	60	
*HSP60*	XM_005894591.1	F: TCGTTTCCTGGGTTCTGCG R: CGGGTAATCGAAGCATTTCTAGG	113	60	
β*-actin*	NM_173979.3	F: GCTGTATTCCCCTCCATCGT R: GGATACCTCTCTTGCTCTGG	97	60	Reference gene
*GAPDH*	NM_001034034.2	F: AGGTCGGAGTGAACGGATTC R: ATGGCGACGATGTCCACTTT	85	60	
*RPL19*	NM_001040516.2	F: CGGAAAAACACCTTGGCTCG R: AGGCTGTGATACATGTGGCG	179	60	

## 2 Materials and methods

### 2.1 Ethics statement and animal preparation

All animal experiments were performed in accordance with the guidelines established by the Gansu Agricultural University Animal Care Committee (2006-398).

Six female yaks were selected, including three yaks from Hezuo City (Hezuo, Gansu, China), at an altitude of approximately 2500 m, and three yaks from Maqu County (Maqu, Gansu, China), at an altitude of approximately 3500 m. The yak were healthy, approximately four years old, and were farmed on a single, large property in the Gannan–Tibetan Autonomous Prefecture (Gansu, China). Five tissues (heart, liver, lung, kidney, and longissimus dorsi) were collected after the yaks were sacrificed. All tissues were snap-frozen in liquid nitrogen and stored at -80 ${}^{\circ }C$ for use in RNA, protein extraction, and enzyme activity assays.

### 2.2 Primer design

Based on the sequence published in GenBank, the primers for *HIF1A*, *PDK4*, *HSP27*, *HSP60*, β *-actin*, *GAPDH*, and *RPL19* gene were designed using the Primer Premier software (version 5.0, PREMIER Biosoft Co., Palo Alto, CA, USA). All primers were synthesized by Beijing AuGCT DNA-SYN Biotechnology Co., Ltd. (Beijing, China). Three housekeeping gene (β *-actin*, accession number: NM_173979.3; *GAPDH*, accession number: NM_001034034.2; *RPL19*, accession number: NM_001040516.2) were used as an internal control to normalize the threshold cycle (Ct) values. The primers are detailed in Table 1.

### 2.3 Quantitative real-time polymerase chain reaction (qPCR) analysis

Tissue RNA was extracted from the five tissues using the Trizol Reagent (Life Technologies, USA) according to the manufacturer's instructions. The quantity and quality of total RNA were monitored using 1.5 % agarose gel electrophoresis and NanoDrop 2000 (Thermo Scientific, USA) instruments. The total RNA extracted from the tissues showed no significant degradation, and the 28S and 18S bands were clearly visible (Fig. S1a).

Reversed transcription to cDNA from RNA was with Evo M-MLV RT Kit (AG, Changsha, China). The cDNA template was used to amplify the housekeeping genes. Three housekeeping genes were well-amplified in five tissues, meeting the requirements for performing qPCR experiments (Fig. S1b). The quantitative real-time polymerase chain reaction (qPCR) reaction system (20 µL) was as follows: total template cDNA 2 µL (100 $\mathrm{ng}\;µL^{-1}$), forward primer 0.4 µL (10 $\mathrm{ng}\;µL^{-1}$), reverse primer 0.4 µL (10 $\mathrm{ng}\;µL^{-1}$), 2× Premix (AG, Changsha, China) 10.4 µL, RNase-Free Water 6.8 µL. The reaction condition was as follows: initial denaturation at 95 ${}^{\circ }C$ for 30 s, denaturation at 95 ${}^{\circ }C$ for 5 s, annealing at 60 ${}^{\circ }C$ for 30 s, and extension at 72 ${}^{\circ }C$ for 30 s (40 cycles). A melting temperature ($T_{m}$) peak at $85\;{}^{\circ }C\pm 0.8$ on the dissociation curve was used to determine the specificity of the PCR amplification, and the melting curves of every genes are only single peaks (Fig. S1c). Based on Ct value of the quantitative expression results, the 2${}^{-\Delta \Delta \text{Ct}}$ method was used to calculate relative expression, the specific calculation formula can refer to the reference (Livak and Schmittgen, 2001).

### 2.4 Western blot analysis

For western blot analysis, three tissues from yak at different altitude were homogenized and lysed using radioimmunoprecipitation assay (RIPA) protein extraction kit (Solarbio, Beijing, China) and phenylmethanesulfonyl fluoride (PMSF) (Solarbio, Beijing, China), according to the manufacturer's instructions. Protein concentrations were quantified using commercial bicinchoninic acid (BCA) protein assay (Vazyme, Nanjing, China). Protein samples were separated by 12 % sodium dodecyl sulfate-polyacrylamide gel electrophoresis (SDS-PAGE) and then transferred onto a polyvinylidene difluoride (PVDF) blotting membrane (Beyotime, Shanghai, China). The membrane was blocked with 5 % non-fat milk in phosphate-buffered saline (PBS) containing Tween-20 (PBST) for 2 h at room temperature and then incubated with rabbit anti-HSP27 polyclonal antibody (1:500; Bioss, Beijing, China), rabbit anti-HSP60 polyclonal antibody (1:500; Bioss, Beijing, China), and rabbit anti-β-actin (loading control) polyclonal antibody (1:5000; Bioss, Beijing, China) at 4 ${}^{\circ }C$ overnight. After being washed with PBST, the membranes were incubated with goat anti-rabbit IgG/horseradish peroxide (HRP) antibody (1:1000; Bioss, Beijing, China) for 2 h at 37 ${}^{\circ }C$. After being washed with PBST, the protein signals were visualized using NcmECL Ultra reagents (New Cell and Molecular Biotech Co. LTD, Suzhou, China) in an X-ray room.

### 2.5 LDH measurement

The LDH levels in heart, lung, and longissimus dorsi tissues were detected using a lactate dehydrogenase assay kits (Nanjing Jiancheng, Nanjing, China). Specifically, 40 mg powdered tissues were added to 360 µL pre-cooled saline (0.9 %) and vortexed for 1 min. The sample was centrifuged at 2000 rpm for 10 min, and the protein concentration of the supernatant was measured using a commercial BCA protein assay (Vazyme, Nanjing, China). To measure LDH levels, 40 µL supernatant, 250 µL matrix buffer, and 50 µL coenzyme 1 solution were combined and incubated at 37 ${}^{\circ }C$ for 15 min in polyethylene tube (5 mL). Then, 250 µL 2,4-dinitrophenylhydrazine was added to the mixture and incubated at 37 ${}^{\circ }C$ for 15 min. Finally, 2.5 mL NaOH (0.4 $\mathrm{mol}\;L^{-1}$) was added and incubated at room temperature for 3 min. A 200 µL volume of the mixture was added to a 96-well plate, and the absorbance values were measured at 440 nm on a plate reader (Multiskan FC; Thermo Fisher Scientific, Beijing, China). LDH contents were calculated as follows: U=(A-B)/(C-D)×E/F, where U was LDH activity in (U $g^{-1}$ protein), A was the absorbance of the sample, B was the absorbance of the control, C was the absorbance of the standard, D was the absorbance of an empty well, E was the concentration of the standard (2 $\mathrm{mmol}\;L^{-1}$), and F was the concentration of the supernatant (g protein ${\mathrm{mL}}^{-1}$). Three biological repeats and two technical replicates were performed.

### 2.6 ATP measurement

The ATP levels in heart, lung, and longissimus dorsi tissues were detected using an ATP assay kit (Nanjing Jiancheng, Nanjing, China). Specifically, 40 mg powdered tissues were added to 360 µL ${\mathrm{ddH}}_{2}O$ and boiled at 98 ${}^{\circ }C$ for 10 min, vortexed for 1 min, and centrifuged at 3500 rpm for 10 min. The protein concentration of the supernatant was measured using a commercial BCA protein assay (Vazyme, Nanjing, China). To measure ATP concentration, 30 µL supernatant, 100 µL substrate |, 200 µL substrate ‖, and 30 µL accelerator were combined in polyethylene tube (5 mL) and incubated at 37 ${}^{\circ }C$ for 30 min. Then, 50 µL precipitant was added, and the mixture was centrifuged at 4000 rpm for 5 min. A volume of 500 µL chromogenic fluids were combined with 300 µL supernatant and incubated at room temperature for 2 min. Finally, 500 µL termination fluids were added and incubated at room temperature for 5 min. A 200 µL volume of the mixture was added to a 96-well plate, and the absorbance values were measured at 636 nm on a plate reader (Multiskan FC; Thermo Fisher Scientific, Beijing, China). The ATP concentration was calculated as follows: Q=(M-N)/(O-P)×R×S/T, where Q was the concentration of ATP ($µ\mathrm{mol}\;g^{-1}$ protein), M was the absorbance of the sample, N was the absorbance of the control, O was the absorbance of the standard, P was the absorbance of an empty well, R was the concentration of the standard ($1\times 10^{3}\;µ\mathrm{mol}\;L^{-1}$), S was the sample dilution ratio before the determination (10×), and T was the concentration of the supernatant (g protein $L^{-1}$). Three biological repeats and two technical replicates were performed.

### 2.7 NADH/NAD${}^{+}$ measurement

The NADH and NAD${}^{+}$ levels in heart, lung, and longissimus dorsi tissues were detected using a NADH/NAD${}^{+}$ assay kit (Beyotime, Shanghai, China) with WST-8. First, the NADH standard was diluted to 6 concentrations: 0, 10, 20, 40, 60, and 80 µM. The absorbance values of the six dilutions were measured. The concentration was plotted along the abscissa, and the absorbance value was plotted along the ordinate to establish a regression equation for the calculation of the NADH and NAD${}^{+}$ concentrations in the tested samples. Then, 30 mg powdered tissues were lysed with 400 µL NADH/NAD${}^{+}$ extract, vortexed for 1 min, and centrifuging at 12000×g for 10 min at 4 ${}^{\circ }C$. To measure NADH, the supernatant was incubated at 60 ${}^{\circ }C$ for 30 min, and 20 µL was added to a 96-well plate. Then, 90 µL of alcohol dehydrogenase was added to the plate and incubated at 37 ${}^{\circ }C$ for 10 min in the dark. Finally, 10 µL chromogenic solutions were added, and the mixture was incubated at 37 ${}^{\circ }C$ for 30 min in the dark. A standard curve was generated and measured at the same time as the samples. The absorbance values were measured at 450 nm and analyzed on a plate reader (Multiskan FC; Thermo Fisher Scientific, Beijing, China). The NAD${}^{+}$ concentration was derived by subtracting the NADH concentration from the total NADH/NAD${}^{+}$ concentration. The regression calculation formula used to determine the NADH/NAD${}^{+}$ content is as follows: Y=0.3013X+0.20 ($R^{2}=0.99$), where Y was the absorbance of the sample and X was the NADH/NAD${}^{+}$ concentration ($µ\mathrm{mol}\;L^{-1}$). Three biological repeats and two technical replicates were performed.

### 2.8 CA measurement

The CA levels in heart, lung, and longissimus dorsi tissues were detected using a CA assay kit (Nanjing Jiancheng, Nanjing, China). Specifically, 100 mg powdered tissues were added to 1000 µL substrate | and vortexed at 0 ${}^{\circ }C$ for 1 min, and centrifuged at 11000g for 10 min. The protein concentration of the supernatant was measured using a commercial BCA protein assay (Vazyme, Nanjing, China). To measure CA levels, 100 µL supernatant, 700 µL substrate |, 100 µL substrate ‖, and 100 µL substrate ∨ were combined in polyethylene tube (2 mL) and incubated at 37 ${}^{\circ }C$ for 30 min. Then, A 200 µL volume of the mixture was added to a 96-well plate, and the absorbance values were measured at 545 nm on a plate reader (Multiskan FC; Thermo Fisher Scientific, Beijing, China). The CA concentration was calculated as follows: Q=(M-N)/(O-P)×R/T, where Q is the concentration of CA ($µ\mathrm{mol}\;g^{-1}$ tissue), M is the absorbance of the sample, N is the absorbance of the control, Q is the absorbance of the standard, P is the absorbance of an empty well, R is the concentration of the standard (0.25 $\mathrm{mol}\;L^{-1}$), and T is the concentration of the supernatant. Three biological repeats were measured.

**Figure 1:**
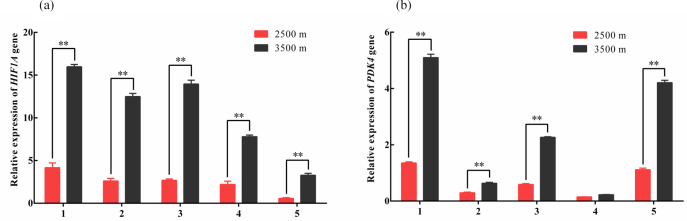
Relative expression levels of *HIF1A* **(a)** and *PDK4* **(b)** mRNA in five tissues obtained from yak living at different altitudes. Note that heart (1), liver (2), lung (3), kidney (4), and longissimus dorsi (5) tissue homogenates were analyzed for mRNA expression. The expression values for the qPCR are given relative to the expression levels of the β *-actin*, *GAPDH*, and *RPL19* gene. The bars represent the mean ± SEM from three independent biological replicates, each performed with three technical replicates. Asterisks denote significant differences: *P<0.05; **P<0.01.

**Figure 2:**
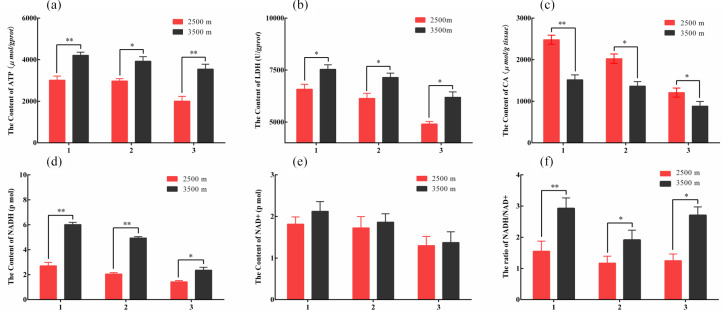
The contents of key glucose metabolism enzyme in various tissues obtained from yak from different altitudes. Note that homogenates from the heart (1), lung (2), and longissimus dorsi (3) tissues were analyzed contents of ATP **(a)**, LDH **(b)**, CA **(c)**, NADH **(d)**, and NAD${}^{+}$ **(e)**. The ratio of NADH/NAD${}^{+}$ **(f)** also was analyzed. The bars represent the mean ± SEM from three independent biological replicates, each performed with three technical replicates. Asterisks denote significant differences: *P<0.05; **P<0.01.

**Figure 3:**
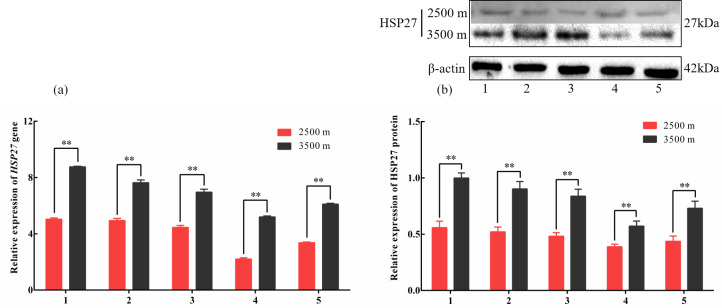
The expression levels of HSP27 mRNA **(a)** and protein **(b)** in five tissues obtained from yak living at different altitudes. Note that heart (1), liver (2), lung (3), kidney (4), and longissimus dorsi (5) tissue homogenates were analyzed for mRNA **(a)** and protein expression **(b)**. The expression values for the qPCR and western blot are given relative to the expression levels of the β *-actin*, *GAPDH*, and *RPL19* gene and β-actin protein. The bars represent the mean ± SEM from three independent biological replicates, each performed with three **(a)** or eight **(b)** technical replicates. Asterisks denote significant differences: *P<0.05; **P<0.01.

### 2.9 Statistical analysis

All figures were generated using GraphPad Prism 6.0 (GraphPad Software Inc, San Diego, CA, US). The band density value of the HSP27 and HSP60 protein was analyzed by AlphaEaseFC image analysis software (AlphaInnotech, USA). Statistical analyses were performed with analysis of variance (ANOVA), followed by Fisher's least significant difference test for multiple comparisons in SPSS 20.0 software (IBM, Armonk, NY, USA). All experimental data are presented as mean ± standard error of the mean (SEM); P<0.05 indicates the difference was significant, and P<0.01 indicates that the different was extremely significant.

## 3 Results

### 3.1 Differences in key glycolysis gene expression between Gannan yaks from different altitudes

To investigate changes in glycolysis-associated gene expression under hypoxic conditions. qPCR was used to measure the expression levels of *HIF1A* and *PDK4* mRNA in the heart, liver, lung, kidney, and longissimus dorsi tissues obtained from yak living at different altitudes. The *HIF1A* and *PDK4* mRNA expression in five tissues from 3500 m were significantly increased compared with those in the tissues from 2500 m yak (P<0.01, Fig. 1a and b), except for *PDK4* expression in kidney. These results indicated that glycolysis might be upregulated under hypoxic conditions in yak from higher altitudes through the increased expression of *HIF1A* and *PDK4*.

### 3.2 Differences in key glycolysis enzyme levels between Gannan yaks from different altitudes

To further investigate the differences in glycolysis between yak from different altitudes. Enzyme assay kits were used to detect the content of LDH, ATP, NADH, NAD${}^{+}$, and CA enzymes of key glucose metabolism levels in the heart, lung, and longissimus dorsi tissues at different altitude yak. The ATP (Fig. 2a), LDH (Fig. 2b), NADH (Fig. 2d), and NAD${}^{+}$ (Fig. 2e) levels were significantly increased in heart, lung, and longissimus dorsi from 3500 m yak compared with those from 2500 m (P<0.05), except for NAD${}^{+}$ levels in three tissues. Significantly decreased of CA level was also observed in three tissues with altitude (P<0.05, Fig. 2c). Further analysis found that the ratio of NADH/NAD${}^{+}$ was significantly increased in three tissues from 3500 m yak than that from 2500 m (P<0.05, Fig. 2f). Taken together, these results supported our hypothesis and indicated that glycolysis might be upregulated and tricarboxylic acid (TCA) cycle was weakened in animals who live at higher altitudes to provide energy under hypoxic conditions.

### 3.3 Divergent HSP response patterns between Gannan yaks from different altitudes

**Figure 4:**
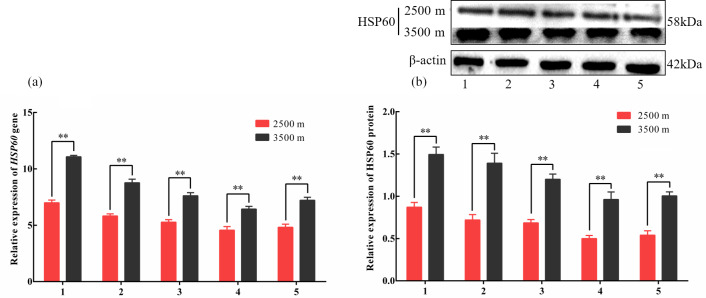
The expression levels of HSP60 mRNA **(a)** and protein **(b)** levels in various tissues obtained from yak living at different altitudes. Note that heart (1), liver (2), lung (3), kidney (4), and longissimus dorsi (5) tissue homogenates were analyzed for mRNA **(a)** and protein expression **(b)**. The expression values for the qPCR and western blot are given relative to the expression levels of the β *-actin*, *GAPDH*, and *RPL19* gene and β-actin protein. The bars represent the mean ± SEM from three independent biological replicates, performed with three **(a)** or eight **(b)** technical replicates. Asterisks denote significant differences: *P<0.05; **P<0.01.

To investigate divergent HSP expression patterns under hypoxic conditions, HSP27 and HSP60 protein and mRNA expression levels were determined by western blotting and qPCR analyses, respectively. The mRNA and protein levels of HSP27, which is an ATP-independent HSP, were significantly increased in five tissues from 3500 m yak compared with those from 2500 m (P<0.05, Fig. 3a and b). In addition, HSP60, an ATP-dependent HSP, demonstrated the same pattern as HSP27, with significantly increased mRNA and protein expression levels in five tissues examined from 3500 m yak compared with those from 2500 m (P<0.05, Fig. 4a and b). Taken together, these results suggested that HSPs were activated in the tissues obtained from yak living at high altitudes under hypoxic conditions. These findings suggested that cytoprotective protein chaperone activity was prioritized over energy conservation in tissues from animals living at high altitudes under hypoxic conditions.

## 4 Discussion

### 4.1 Glycolysis is upregulated in Gannan yaks during hypoxia

In the present study, the glycolysis response was examined in adaptation to hypoxia in tissues taken from yak from different altitudes for the first time. This study found that the expression of *HIF1A* and *PDK4* gene in the tissue was positively correlated with the altitude level, that is, the higher expression of *HIF1A* and* PDK4* at higher altitude level. In human cancer research, cells are often exposed to hypoxia due to the competitive growth of tumors (Gatenby and Gillies, 2004). A study by Rellinger et al. (2015) in neuroblastoma cells found that gastrin-releasing peptide receptor (GRP-R) regulates glucose metabolism in neuroblastoma cells by modulating *HIF1A* and *PDK4* expression. *PDK4* regulates glucose metabolism, in part, through the regulation of *HIF1A* (Rellinger et al., 2015). This is consistent with the results of our study. We found that *HIF1A* and *PDK4* displayed similar patterns in various tissues which reflects the close positive relationship between *HIF1A* and *PDK4* during the regulation of glucose metabolism at high altitude level. Zhu et al. (2018) found that the stabilization or nuclear translocation of *HIF1A* increased *PDK4* expression when studying diabetes mellitus (Zhu et al., 2018). Previous studies have demonstrated that the phosphoinositide 3-kinase (PI3K)/Akt pathway becomes activated under hypoxic conditions, resulting in changes in the activities of various enzymatic biological pathways, including glucose metabolism (Xie et al., 2019). *HIF1A* has been demonstrated to be a glycolysis promoter that is regulated by PI3K/Akt pathway, and increased *HIF1A* expression induces the transcription of *PDK4* (Kaelin and Ratcliffe, 2008; Lambert et al., 2010; Idelevich et al., 2011). *PDK4* can phosphorylate the pyruvate dehydrogenase complex, inhibiting the oxidative decarboxylation of pyruvate into acetyl-CoA. Therefore, we suspect that the reason may be that the increase of glycolysis causes the *HIF1A* and *PDK4* high expressed at high altitude level.

We also found that the key enzymes level of glycolysis was positively correlated with the altitude level, that is, the NADH, ATP, NAD${}^{+}$ and LDH content were higher in the tissues with higher altitude. Wei et al. (2016) found that LDH contents increased with increased glycolysis levels and were previously demonstrated to be expressed at high levels in the high-altitude plateau pika (Wei et al., 2016). Mohammed et al. (2015) found that hypoxic conditions resulted in increased LDH, lactate, and NAD${}^{+}$ levels when mice were exposed to either 21 % (control) or 18 % (mild hypoxia) oxygen conditions for 24 h (Mohammed et al., 2015). Compared to the content of NADH, ATP, LDH, the ratio of NADH/NAD in the same tissue was significantly higher in 3500 m yak than in 2500 m and with the highest levels observed in the heart tissues. These results suggesting that the 3500 m yak had higher glycolysis level to maintain energy supply and reflect the increased oxygen sensitivity of the heart compared with other tissues and the need to ensure an adequate energy supply for the heart to sustain life. Similar observations have been reported in human cancer. Koka et al. (2018) found only negligible LDH activity in cancer stem cells (CSCs) under normoxic conditions, whereas CSCs exhibited the increased production of ATP and LDH under hypoxic conditions (Koka et al., 2018). In our study, we also found that CA was significantly decreased with altitude. We suspect that the reason may be that the increase of altitude causes the inhibition of TCA cycle to reduce oxygen consumption. Tang et al. (2019) found that hypoxia promotes the growth of human breast tumorigenic cells, which dependent on the attenuated TCA cycle (Tang et al., 2019). Therefore, the increase of anaerobic glycolysis may also be the result of weakness TCA cycle on glucose metabolism, which can reduce oxygen consumption and ensure energy supply at hypoxia environment. This unique mechanism may help yak better adapt to high-altitude environments.

### 4.2 HSPs are activated to maintain proteostasis in Gannan yaks during hypoxia

As briefly discussed in the introduction, in addition to ensuring a sufficient energy supply, an alternative means of adaptation to hypoxic conditions is the maintenance of proteostasis through the activation of HSPs. In the present study, HSP27 and HSP60 gene and protein expression levels were globally increased in various tissues obtained from yak living in hypoxic conditions. It indicated that living in hypoxic conditions did not change the HSP expression pattern with respect to either the functional classification or ATP dependency of HSPs. HSP27 is an ATP-independent holdase, which serves as a co-chaperone that recognizes and stabilizes unfolded proteins and delivers them to foldases for refolding (Díaz et al., 2015). Because HSP27 does not require ATP, its functions are presumably maintained during prolonged exposure to hypoxic conditions. Therefore, the initial hypoxic trigger results in the activation of HSP27, and the gene and protein expression levels observed in the current study suggested that HSP27 cytoprotective protein chaperone activity is activated in yak living under hypoxic conditions. Protein synthesis represents one of the most energetically expensive cellular processes (Stouthamer, 1973). However, we found that the ATP contents increased significantly under hypoxic conditions; therefore, the ATP required for HSP27 folding is negligible relative to the ATP required for the whole body. During acute hypoxia, HSP27 expression was found to decrease, despite being an ATP-independent HSP (Nguyen et al., 2019).

HSP60 is classified as an ATP-dependent foldase chaperone protein, which actively refolds unfolded proteins via ATP hydrolysis (Díaz et al., 2015). Because HSP60 requires ATP to function, the downregulation of this protein in an ATP-deficient environment, such as hypoxia, would contribute to the conservation of energy for other, more important, cellular processes (Xu, 2018). However, we found that the HSP60 gene and protein expression levels were significantly increased in tissue from yak living under hypoxic conditions. The observed upregulation of glycolysis in animals living under hypoxic conditions would ensure the sufficient availability of ATP for HSP function, which is supported by the increased ATP contents observed in tissues obtained from HA animals. Similar to our results, Li et al. (2019) found that HSP60 expression levels in rats rapidly increased when exposed to high-altitude, hypoxic environmental conditions (Li et al., 2019). One study found that hypoxia-tolerant hard clams and oysters were able to maintain the steady-state activity levels of both ATP-dependent and ATP-independent mitochondrial proteases under both anoxia and 5 % $O_{2}$ conditions (Ivanina and Sokolova, 2016). Our results showed a similar expression trend, with significantly increased gene and protein levels observed for both HSP27 and HSP60 in tissues obtained from yak living under high-altitude, hypoxic conditions compared with those in tissues obtained from yak living at lower altitudes. In support of our hypothesis, HSP27 and HSP60 levels were significantly increased in tissues from animals living under hypoxic conditions, regardless of their functional dependence on ATP. This mechanism would facilitate the maintenance of proteostasis in cells under hypoxic conditions. Our findings suggested that the upregulation of glycolysis ensures an adequate energy supply, while the upregulation of HSPs ensures the homeostasis of intracellular proteins, allowing the maintenance of normal physiological functions. These molecular chaperones both enhance yak proteasome activities and preserve proteasomal functions under stressful conditions, although the specific roles played by these pathways during hypoxia have yet to be investigated.

## 5 Conclusions

In summary, this study found that hypoxia-reprogrammed glucose metabolism promotes energy supply via up-regulated glycolysis and weakness of TCA cycle in Gannan yaks, which ensure the availability of an adequate energy supply and reduce oxygen consumption. HSPs become activated to ensure the maintenance of protein homeostasis, allowing the yak's body to perform normal physiological functions under hypoxic conditions.

## Supplement

10.5194/aab-64-345-2021-supplement The supplement related to this article is available online at: https://doi.org/10.5194/aab-64-345-2021-supplement.

## Data Availability

The data sets used in this paper are available from the corresponding author upon request.
