# Supplementary material for: Regulating glycolysis and heat shock proteins in Gannan yaks (Bos grunniens) in response to hypoxia of the Qinghai–Tibet Plateau
Source: Arch Anim Breed. 2021 Aug 19;64(2):345–53. doi: 10.5194/aab-64-345-2021 (PMC8386194; doi:10.5194/aab-64-345-2021)
Supplement: The supplement related to this article is available online at: https://doi.org/10.5194/aab-64-345-2021-supplement. [file aab-64-345-supplement.zip › aab-64-345-2021-supplement-title-page.pdf]

Supplement of Arch. Anim. Breed., 64, 345–353, 2021
https://doi.org/10.5194/aab-64-345-2021-supplement
© Author(s) 2021. CC BY 4.0 License.

Open Access
Archives Animal Breeding

Supplement of

Regulating glycolysis and heat shock proteins in Gannan yaks (*Bos grunniens*) in response to hypoxia of the Qinghai–Tibet Plateau

Yuliang Wen et al.

Correspondence to: Jiang Hu (huj@gsau.edu.cn) and Yuzhu Luo (luoyz@gsau.edu.cn)

- aab-64-345-2021-supplement-title-page.pdf
- Figure S1.tif

The copyright of individual parts of the supplement might differ from the article licence.
