# Supplementary figures and images for: Regulating glycolysis and heat shock proteins in Gannan yaks (Bos grunniens) in response to hypoxia of the Qinghai–Tibet Plateau
Source: Arch Anim Breed. 2021 Aug 19;64(2):345–53. doi: 10.5194/aab-64-345-2021 (PMC8386194; doi:10.5194/aab-64-345-2021)

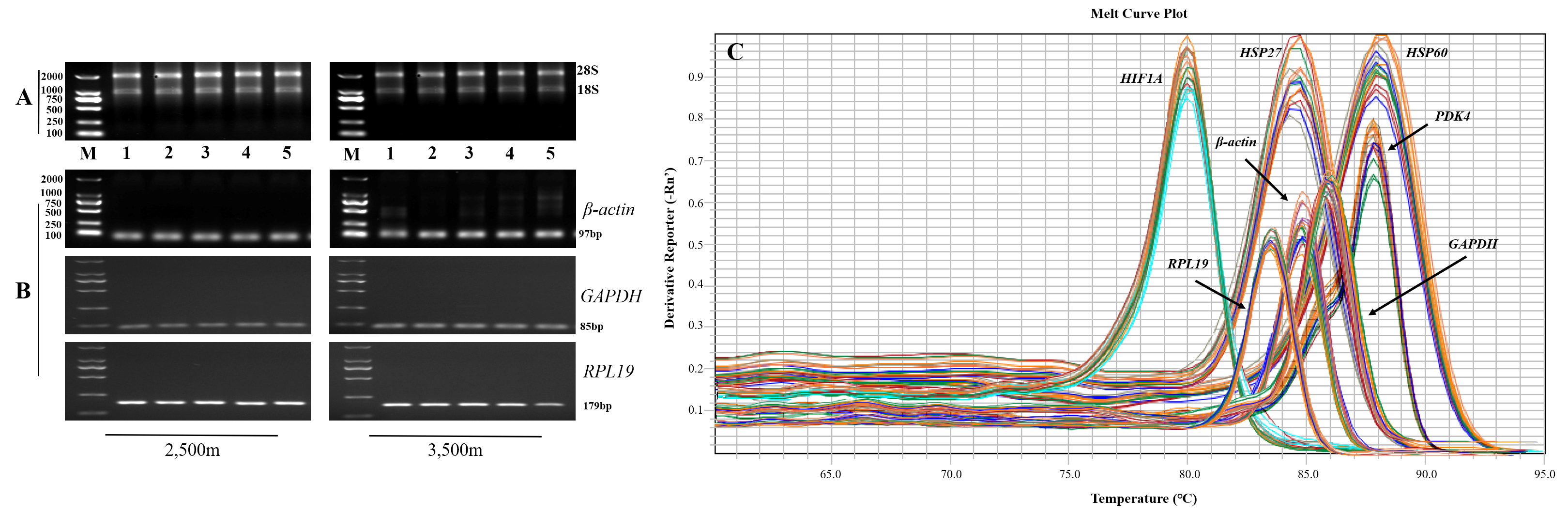

Supplement: The supplement related to this article is available online at: https://doi.org/10.5194/aab-64-345-2021-supplement. [file aab-64-345-supplement.zip › Figure S1.tif]
